# Decoding the germline genetic architecture of prostate cancer at a single cell resolution

**DOI:** 10.1371/journal.pgen.1011975

**Published:** 2025-12-30

**Authors:** Cheng Wang, Tianqi Tang, Yuejun Wang, Jingjing Li

**Affiliations:** 1 Eli and Edythe Broad Center of Regeneration Medicine and Stem Cell Research, University of California San Francisco, San Francisco, California, United States of America; 2 Bakar Computational Health Sciences Institute, University of California San Francisco, San Francisco, California, United States of America; 3 Center for Reproductive Science, School of Medicine, University of California San Francisco, San Francisco, California, United States of America; 4 Department of Neurology, School of Medicine, University of California San Francisco, San Francisco, California, United States of America; The University of Texas Southwestern Medical Center Eugene McDermott Center for Human Growth and Development, UNITED STATES OF AMERICA

## Abstract

Prostate cancer exhibits a strong familial association, and its heritability indicates a significant contribution from germline variants. While genome-wide association studies (GWAS) have identified common germline variants associated with prostate cancer risk, translating these statistical associations into functional mechanisms has remained a long-standing challenge. Consequently, most of our understanding of the genetic basis of prostate cancer stems from extensive studies of somatic mutations, leaving the germline genetic architecture largely unresolved. Because most germline variants lie in the noncoding genome and complex human diseases are predominantly driven by regulatory mutations, we herein asked which prostate cell types mediate the functional effects of germline variants, and thus represent the most genetically vulnerable populations. We generated paired epigenomic and transcriptomic profiles from reference human prostate tissues. Integrating these single-cell data with large-scale GWAS data identified a terminally differentiated luminal epithelial subtype that mediates the strongest germline risk in prostate cancer. We subsequently developed a deep learning model to score ~17 million GWAS variants based on their predicted impact on altering local chromatin accessibility in this vulnerable luminal epithelial subtype, and identified high-confidence candidate loci where high-risk germline variants likely alter promoter accessibility in prostate cancer. The implicated genes were involved in several pathways in tumorigenesis, displayed strong dosage sensitivity, and converged on the androgen receptor (AR)-mediated regulon, a mechanism also observed for somatic mutations. Overall, by unveiling cell types and candidate loci that mediate germline risk, our study defines the cell-type-specific germline architecture in prostate cancer and provides a comprehensive framework for understanding cancer heritability.

## Author summary

Prostate cancer tends to run in families, suggesting that inherited genetic factors strongly influence a man’s risk of developing the disease. Although large-scale genetic studies have identified many variants associated with prostate cancer, most of these variants lie outside genes, in the noncoding regions of the genome, making it difficult to determine how they function. To address this challenge, we studied healthy human prostate tissue at single-cell resolution to identify which cell types are most affected by inherited genetic variation. By combining single-cell gene expression and chromatin accessibility with population-level genetic data, we found that a specific subtype of luminal epithelium carries the strongest inherited risk for prostate cancer. We then developed a deep learning model, DEEP + , to predict how millions of common genetic variants influence gene regulation in this cancer-relevant cell type. Our findings highlight key genetic regions and genes that affect cancer-related pathways and androgen receptor (AR) signaling, providing new insight into how inherited variants shape prostate cancer susceptibility.

## Introduction

Prostate cancer is a leading malignancy among men in the United States, accounting for more than a quarter of all cancer diagnoses and ranking among the top causes of cancer-related mortality in men [1]. Its pronounced heritability (estimated at approximately 57%) [2] underscores a substantial genetic component, suggesting that germline variants contribute significantly to its molecular underpinnings. Although genome-wide association studies (GWAS) have identified a number of these germline variants [3–14], the mechanistic details remain elusive, leaving the broader germline genetic architecture of prostate cancer largely uncharacterized. By contrast, our current understanding of the disease’s genetic basis has come from studies of somatic mutations [15].

One key limitation of GWAS is their tendency to pinpoint sentinel variants marking disease-associated haplotypes without offering a clear path to identifying the causal alleles. Therefore, many biologically relevant variants remain obscured within sets of statistically associated loci. Furthermore, common variants often exhibit modest effect sizes, which cannot be effectively captured by conventional GWAS relying on a uniform genome-wide significance threshold. This challenge is particularly acute in prostate cancer, where risk is not attributed to a few dominant loci but instead emerges from a highly polygenic architecture involving the aggregated effects of numerous loci across the genome. Without bridging the gap between statistical association and functional mechanism, our understanding of how germline variation drives prostate cancer risk will remain incomplete.

Our recent analyses of significant prostate cancer GWAS loci listed in the NHGRI-EBI GWAS Catalog [16] revealed their predominant localization to noncoding human genome. This pattern aligns with growing evidence that regulatory variants play a pivotal role in complex diseases [17,18]. However, such regulatory variants further complicate the search for functional alleles: because gene regulation is both tissue- and cell-type-specific, potentially pathogenic germline variants cannot be solely inferred from genomic sequences but need to be functionally characterized in the appropriate cellular context.

The prostate gland is distinguished by its remarkable cellular heterogeneity, with glandular acini lined by luminal epithelial (LE) cells that mediate androgen signaling. These LE cells are derived from basal epithelial (BE) cells that form a protective progenitor layer beneath them [19]. Beyond the epithelial compartment, the functional integrity of the prostate and its dynamic responsiveness to hormonal cues are sustained through intricate interactions with stromal, smooth muscle, and immune cells. Despite substantial mutational heterogeneity across patients, genetic and molecular alterations in prostate cancer are convergent onto dysregulated androgen receptor (AR) signaling [20–24]. In the normal prostate, the AR activation by androgens, such as testosterone and dihydrotestosterone, enables it to function as a transcription factor regulating genes involved in cell proliferation, differentiation, and survival. In prostate cancer, alterations in AR signaling promote tumor initiation and progression [21]. While AR is highly expressed in luminal epithelial cells and the prostate stroma, their respective contributions to prostate cancer etiology remain elusive. More importantly, human luminal epithelial cells display substantial cellular heterogeneity [25], naturally raising the question of which specific cell state(s) or subtype(s) contribute the most to prostate cancer risk.

**Fig 1:**
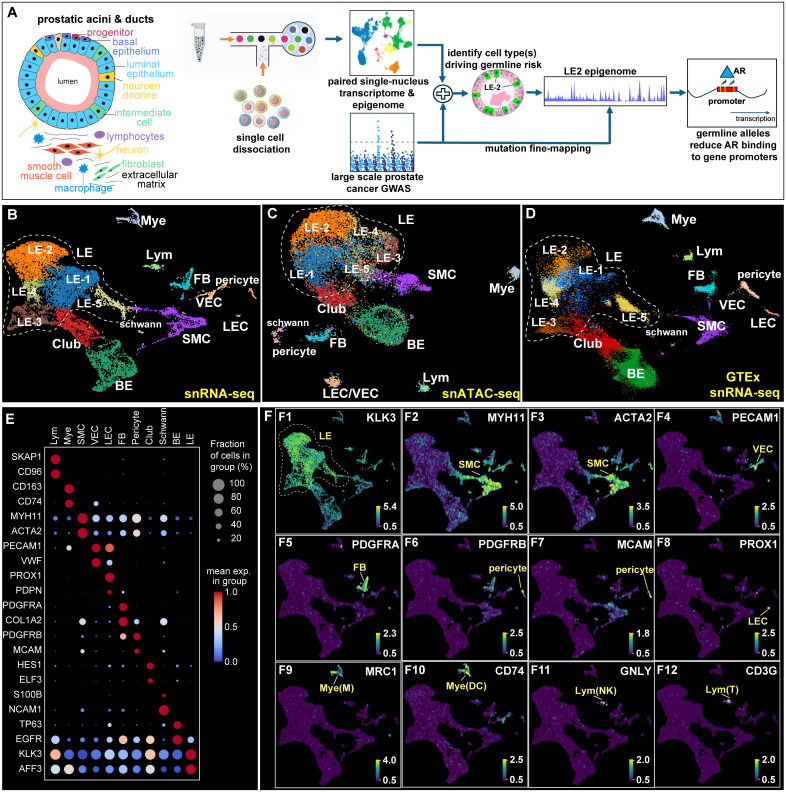
Integrating single-cell multiomics to uncover germline risk in prostate cancer. **(A)** Schematic overview of the study: Normal prostate gland tissues were analyzed using 10X Chromium snMultiome to jointly profile snRNA-seq and snATAC-seq. Single-cell data integration with large-scale prostate cancer GWAS identified a luminal epithelial subtype (LE-2) as the primary mediator of germline risk. Fine mapping of germline variants in LE-2 highlighted high-confidence risk alleles disrupting androgen receptor (AR) binding, predisposing individuals to elevated prostate cancer risk. **(B-D)** UMAP projections of transcriptomic **(B)** and epigenomic **(C)** profiles of normal prostate tissue, with cell types/subtypes identified via label transfer from GTEx data **(D)**, revealing cellular diversity. **(E)** Dot plot displaying marker gene expression profiles across 11 cell types. Dot size and color intensity represent the fraction of cells expressing each gene and average expression, respectively. (**F1-F12**) Marker gene expression visualized on the snRNA-seq UMAP. Abbreviations: LE (luminal epithelium), SMC (smooth muscle cells), VEC (vascular endothelial cells), FB (fibroblasts), Mye (myeloid cells), M (macrophages), DC (dendritic cells), Lym (lymphocytes), NK (natural killer cells), T (T lymphocytes).

Given the pronounced heritability of prostate cancer [2], we herein asked which prostate cell types mediate the strongest germline risk in prostate cancer. Since most germline variants fall in the noncoding genome, contributing to disease through dysregulating gene expression, we set out to map, for the first time, the germline genetic architecture of prostate cancer across cellular subtypes of the prostate. By defining the cell populations in which risk variants operate, we aimed to reveal the core pathways disrupted by germline alleles and how molecular and cellular vulnerability shapes inherited cancer risk. To achieve this, we performed single-cell multiomic profiling of normal human prostate tissues, generating paired reference epigenomes and transcriptomes in single cells (**Fig 1A**). This approach allowed us to identify distinct cell states and subtypes, facilitating mapping disease-associated variants onto the reference single cell map. Integrating GWAS data, our study uncovered a specific luminal epithelial subtype that mediates the strongest germline risk in prostate cancer. Subsequent deep learning analysis revealed high-confident loci affecting the gene regulatory landscape within this luminal lineage, converging on a FOXA1/AR-mediated regulon. Notably, this germline-driven convergence onto FOXA1/AR pathways parallels the well-established somatic mutations that also funnel into AR-regulated networks, underscoring a shared molecular axis of prostate cancer pathogenesis. These insights underscore that the germline architecture of prostate cancer risk is both functionally coherent and cell-type-specific, carrying significant implications for clinical practice. Identifying the cell types and genes that are most genetically vulnerable is expected to refine risk stratification, guide the development of targeted interventions, and ultimately improve patient outcomes. As the genetic foundation of prostate cancer comes into sharper focus, leveraging these insights for improved diagnosis, prevention, and treatment becomes increasingly essential.

## Results

### Constructing the reference single-cell multiomics dataset in the human prostate

Because complex diseases are primarily driven by noncoding regulatory mutations, we began our investigation by examining germline variants from a large cohort of prostate cancer patients. Our goal was to determine whether at-risk alleles are preferentially enriched within active epigenomic regions of specific prostate cell types. For the cell types found to be (epi)genetically vulnerable, we then performed a genome-wide scan to quantify how individual germline alleles alter cell-type-specific gene regulation (**Fig 1A**). Central to this approach is the construction of a reference regulatory landscape for each cell type in healthy prostate tissue for downstream fine mutation mapping. Establishing such a baseline dataset allows us to evaluate how at-risk alleles shift normal gene regulatory programs toward a disease-prone state.

We performed single-cell profiling in reference prostate samples from healthy individuals obtained from the ENCODE consortium and characterized cell-type-specific gene regulation and expression. Using the 10X Genomics single-nucleus multiomic platform (Chromium Single Cell Multiome ATAC + Gene Expression), we profiled the paired ATAC-seq (the open chromatin epigenome) and RNA-seq (the transcriptome) data for total 19,182 nuclei from human prostate tissues. After stringent quality control (S1 Table), we retained 12,020 high-quality single cells with matched RNA-seq and ATAC-seq profiles. We performed clustering analyses in both the epigenomic (Fig 1B) and transcriptomic (Fig 1C) spaces. To optimize the number of clusters, we systematically varied clustering resolution and evaluated two metrics—silhouette score (cluster cohesion) and within-cluster inertia (cluster compactness) (S1A-S1B Fig) (Methods and Materials). The optimal parameters yielded 15 distinct clusters representing the major prostate cell types. To confirm biological relevance, we applied label transfer to align our single-cell transcriptomes with those from healthy prostate samples in the GTEx cohort [26]. The resulting one-to-one correspondence between clusters (**Fig 1D**) confirmed that our dataset recapitulates the canonical prostate cellular landscape at the population level. Because GTEx provides only transcriptomic without matched epigenomic data, we used our dataset as the reference for subsequent fine-scale variant mapping.

Using curated cell-type markers, we unambiguously annotated each cluster to a specific prosate cell type (**Fig 1E**). The concordance between epigenomic and transcriptomic cluster identities suggested that prostate cell types are primarily defined at the epigenomic level and manifested at the transcriptomic level. We systematically examined cell-type–specific marker expression: as expected, luminal epithelial (LE) cells were readily identified by the prostate-specific antigen *KLK3* (PSA) (**Fig 1F1**). Smooth muscle cells displayed canonical markers *MYH11* and *ACTA2*, whereas vascular endothelial cells were distinguished by *PECAM1*(CD31), and lymphatic endothelial cells were marked by *PROX1*. Fibroblasts were characterized by *PDGFRA* expression, and perivascular cells were marked by *PDGFRB* and *MCAM*. Among immune-associated cell types, myeloid populations included macrophages (*MRC1*^*+*^) and dendritic cells (*CD74*^*+*^), and lymphoid populations encompassed NK cells (*GNLY*^+^) and T cells (*CD3G*^+^) (**Fig 1F**). Overall, these observations underscore the high quality of our dataset and confirm that our single-cell profiling captures the expected repertoire of cell types present in the normal human prostate gland.

### Dissecting luminal epithelium into five distinct cell subtypes/states

**Fig 2:**
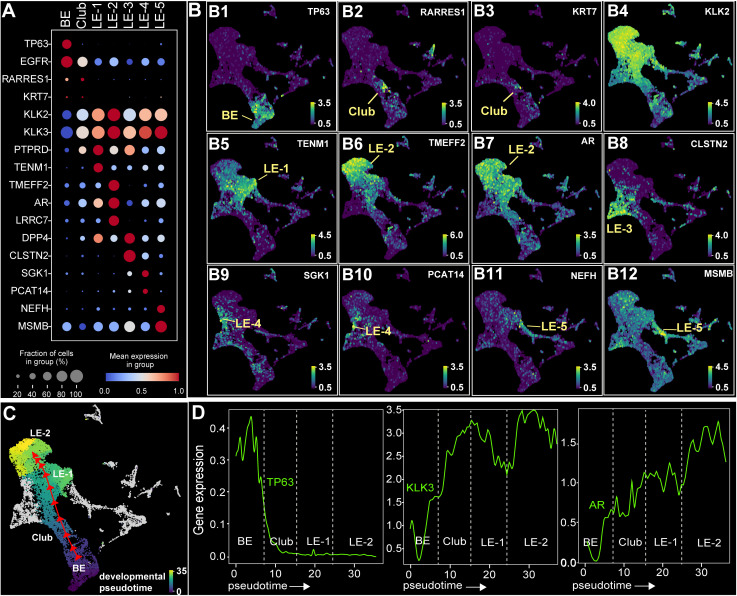
Molecular characteristics of luminal epithelial cell subtypes. **(A)** Dot plot highlighting marker genes that define BE (basal epithelium), club cells, and five LE subtypes (LE-1–LE-5). **(B1-B12)** Specific expression of LE subtype markers on the single cell transcriptome UMAP. **(C)** Differentiation trajectory reconstructed from BE cells to LE-2, with pseudotime indicating cell developmental progression. Arrow marks the primary differentiation direction. **(D)** Expression dynamics of *TP63*, *KLK3*, and *AR* during transitions from BE to LE-2, showing *TP63* down-regulation and *KLK3/AR* up-regulation along the developmental trajectory.

We observed notable heterogeneity within the epithelial compartment, extending from basal epithelial (BE) cells and club cells to five distinct luminal epithelial (LE) states or subtypes (LE-1–LE-5; **Fig 1B**, **1C**). The presence of these subtypes in the independent GTEx dataset (**Fig 1D**) confirmed that they are representative components of normal prostate tissue. To further elucidate their molecular identities, we performed differential gene expression analyses. Aligning these results with established epithelial markers, we annotated each subtype and demonstrated their distinct transcriptomic profiles (**Fig 2A**). As expected, BE cells were uniquely marked by the known marker *TP63*, whereas club cells were distinguished by *RARRES1* or *KRT7* (**Fig 2A**, **2B2, 2B3**). Club cells also displayed heterogeneity based on a few recently suggested markers like *CP*, *WFDC2* and *PIGR* [27,28] (S2A-S2C Fig). We noted specific expression of *ELF3* in club cells (S2D Fig).

Among the LE subtypes, LE-1 cells displayed progenitor-like characteristics by specifically expressing *TENM1* (teneurin transmembrane protein 1), which has been implicated in prostate cancer differentiation [29]. LE-2 cells specifically expressed *TMEFF2* (**Fig 2B6**), which is an androgen-dependent suppressor of prostate cancer cell growth. Interestingly, androgen receptor (*AR*) expression was strongest and most specific in the progenitor-like LE-1 and the more differentiated LE-2 subtypes, while its expression was moderate in other LE subtypes (**Fig 2B7**). This observation suggests significant roles for LE-1 and LE-2 in mediating *AR*-dependent functions. In sharp contrast to LE-1 and LE-2, LE-3 cells were specifically marked by *DPP4* (Dipeptidyl peptidase 4), an AR-stimulated tumor suppressor in prostate cancer. In parallel, LE-4 cells were uniquely characterized by *SGK1* (glucocorticoid-induced protein kinase 1) (**Fig 2B9**), which is upregulated in primary prostate tumors [30], as well as the well-established RNA gene *PCAT14* (*prostate cancer associated transcript 14*, **Fig 2B**), a biomarker for prostate cancer diagnosis [31] and response to androgen-deprivation therapy [32]. LE-5 cells, while expressing common luminal markers such as *KLK3*, exhibited a more divergent transcriptome compared to LE-1–4 (**Fig 2B**). Notably, LE-5 specifically expressed *NEFH* and *MSMB*. *NEFH* functions as a tumor suppressor [33], while *MSMB* encodes a prostate-secreted protein and biomarker for prostate cancer [34]. Both *NEFH* and *MSMB* are downregulated in prostate cancer [34,35]. These observations collectively suggest that LE-1–5 subtypes are pervasively involved in prostate cancer, each contributing distinct features to disease pathogenesis.

As LEs are developmentally derived from BEs, we leveraged the single-cell transcriptomic data to infer cell developmental trajectories. We reconstructed the differentiation continuum from BE, club cells, the progenitor-like LE-1 to the mature LE-2 subtype (**Fig 2C**). Though the precise developmental origin of the club cell remains unresolved, our analysis suggested a transitioning nature of club cells and LE-1 subtype, and confirmed that LE-2 represents a terminally differentiated LE subtype. Along the LE-2 maturation trajectory, we observed a progressive decline in the BE marker *TP63*, accompanied by a steady increase in the PSA gene *KLK3* and *AR* along cell development progression (**Fig 2D**). Notably, LE-3/4 and LE-5 were in divergent developmental trajectories from LE-2, suggesting distinct branches of the cell fates (S2E-S2F Fig).

### Constructing single cell germline risk map for prostate cancer

After generating paired single-cell chromatin epigenomes and transcriptomes from our reference prostate tissues, we next aimed to identify the cell types most strongly associated with germline genetic risk for prostate cancer. To this end, we leveraged a large-scale prostate cancer genome-wide association study (GWAS) dataset comprising 79,194 cases and 61,112 controls of European ancestry [10]. This dataset provided summary statistics (including risk Z-scores) for approximately 17 million germline single nucleotide polymorphisms (SNPs). To integrate these GWAS results with our single-cell profiles, we applied the SCAVENGE algorithm [36]. Briefly, SCAVENGE aggregates the GWAS risk Z-scores of SNPs located within each cell’s active regulatory elements (i.e., open chromatin regions), conceptually generating a chromatin-informed polygenic risk score for each cell. Because each cell exhibits a unique epigenomic configuration, the resulting aggregated risk associations differs from cell to cell. However, cells belonging to the same cell type share highly similar epigenomic landscapes, and therefore tend to exhibit similar genetic risk. Thus, by comparing SCAVENGE scores across single cells and across cell types, we can pinpoint cell type(s) most vulnerable to germline genetic risk for prostate cancer.

**Fig 3:**
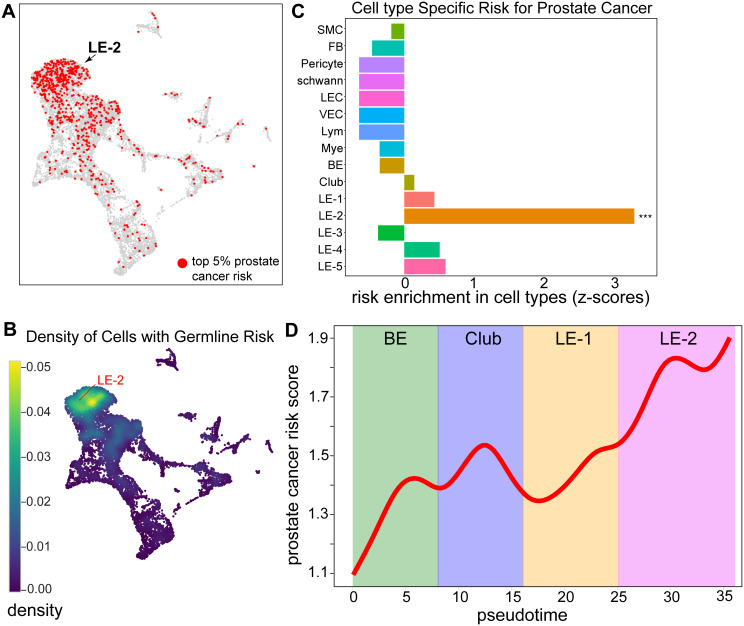
Single-cell germline risk map in prostate cancer. **(A, B)** Integrative analysis of single-cell multiomics and GWAS data identified vulnerable cells (highlighted in red) to germline risk in prostate cancer. These vulnerable cells (top 5% in red, **A**) were predominantly concentrated in the LE-2 subtype. Nebulosa plot showing the density of vulnerable cells on the UMAP **(B)**. **(C)** Statistical permutation analysis confirmed that the LE-2 cell subtype was the only cell population significantly associated with germline risk in prostate cancer. *** denotes p < 0.001 (permutation test). Risk enrichment was assessed by Z-scores indicating the enrichment of genetically vulnerable cells in each cell type. **(D)** Grouping cells into pseudotime bins along the developmental trajectory from basal epithelium (BE) to the terminally differentiated LE-2 subtype revealed a progressive elevation of germline risk along the cell developmental trajectory.

We applied SCAVENGE to integrate our single-cell multiomics data with large-scale prostate cancer GWAS results, generating a single-cell germline risk map (**Fig 3A**, **3B**). Notably, cells exhibiting significant prostate cancer risk associations were concentrated within the LE-2 subtype, a striking contrast to their relative depletion in non-LE cell types (**Fig 3A**, **3B**). This observation was statistically validated at the cell-type level, where LE-2 was the only subtype displaying a significant risk association with prostate cancer (**Fig 3C**). In other words, the active gene regulatory elements defining the LE-2 subtype are strongly enriched for high-risk germline variants. Moreover, we examined the developmental trajectory from BE to LE-2 (**Fig 2C**). Grouping cells by pseudotime intervals revealed a progressive increase in germline risk, culminating at the terminally differentiated LE-2 state (**Fig 3D**). Notably, the risk increase is accompanied by the increasing AR expression during LE-2 maturation, suggesting a regulatory convergence between germline risk and AR-driven transcriptional programs (**Fig 2D**). Taken together, our analysis, for the first time, demonstrated that germline genetic risk for prostate cancer is mediated by the terminally differentiated LE-2 cells in the human prostate.

### Developing a deep learning model for fine mutation mapping in LE-2 cells

We next sought to identify potentially functional germline alleles in LE-2, with the goal of revealing key molecular pathways rendered vulnerable to germline risk, thereby predisposing individuals to prostate cancer. Given the strong enrichment of high-risk variants in the LE-2-specific epigenome (**Fig 3B**), we scanned all ~ 17 million SNPs from the same prostate cancer GWAS cohort to evaluate the regulatory effects of each allele on perturbing local chromatin accessibility in LE-2 cells.

**Fig 4:**
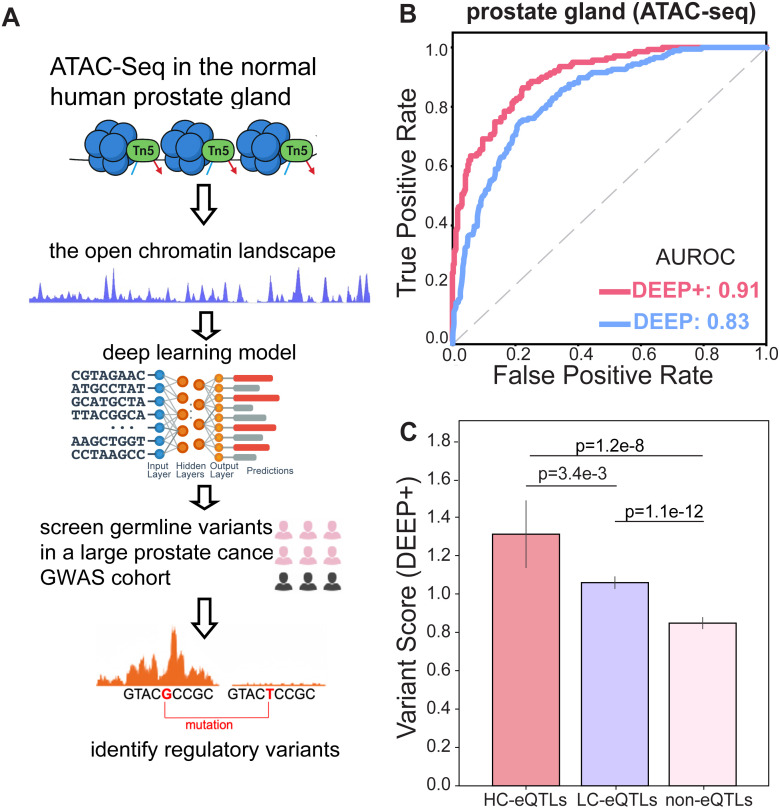
A deep learning framework for fine mutation mapping in LE-2 cells. **(A)** A deep learning framework was trained to predict chromatin accessibility from genomic sequences. The trained model was then applied to screen the genome, enabling the identification of allelic effects on local chromatin accessibility. **(B)** The enhanced DEEP+ model outperformed the previously developed DEEP model, demonstrating improved accuracy in predicting chromatin accessibility. **(C)** Validation using GTEx prostate eQTL data showed that high-confidence eQTLs (HC-eQTLs) were consistently assigned the highest scores by DEEP + , followed by low-confidence eQTLs (LC-eQTLs) and non-eQTL loci. These results validated the model by demonstrating that loci identified by DEEP+ as having regulatory effects are more likely to impact gene expression.

To achieve this, we employed our recently developed deep learning framework, DEEP (Deep Estimation from Epigenome Prediction) [37,38], which was originally designed to identify regulatory somatic alleles in prostate cancer. As outlined in **Fig 4A**, the model operates in two sequential stages: [1] It is first trained on human genomic sequences encompassing ATAC-seq peaks from tissues or cell types of interest and is subsequently validated in blind tests to ensure robust prediction of chromatin accessibility for any given genomic sequence. [2] Once trained, the model is used to score genomic sequences containing candidate variants by estimating the chromatin accessibility for both the reference and alternative alleles. The difference between these predictions reflects the allelic impact on local chromatin structure, where positive and negative scores, respectively, indicate increased and decreased local chromatin structure for a given allele. In this study, we refined our original DEEP model [35,36] by introducing the DEEP+ framework, which replaces the convolutional neural network (CNN) backbone with a residual neural network architecture (S3 Fig). Since DEEP was initially trained on ATAC-seq data from bulk prostate tissues (from ENCODE) [39], we adopted the same training strategy and dataset for DEEP + . The updated DEEP+ model outperformed the original DEEP model in predicting prostate chromatin accessibility on previously unseen genomic sequences (**Fig 4B**). To ensure that the chromatin-altering alleles identified by DEEP+ also influence gene expression, we applied the trained DEEP+ model to score 356,598 common variants from the GTEx project [40]. We observed that the prostate eQTLs identified by GTEx were consistently received significantly higher DEEP+ scores (**Fig 4C**), confirming that regulatory alleles identified by DEEP+ have a significant impact on gene expression in prostate tissue.

### Functional characterization of LE-2-specific germline regulatory alleles in prostate cancer

**Fig 5:**
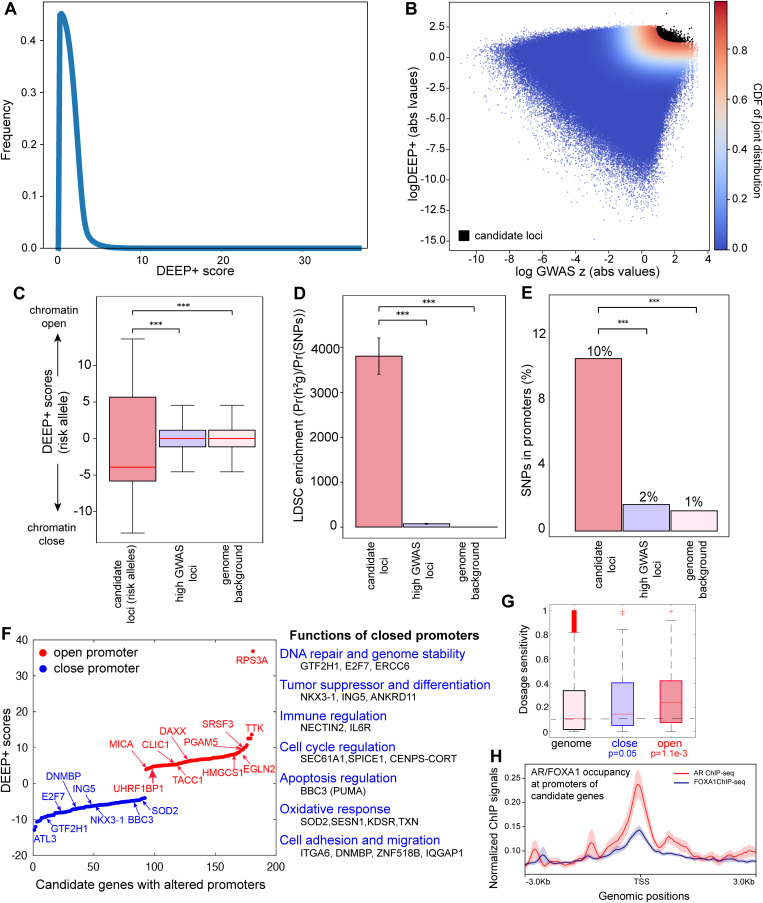
Functional characterization of high-confidence germline alleles in prostate cancer. **(A)** Screening ~17 million SNPs in the prostate cancer GWAS dataset using DEEP+ identified an excess of alleles with strong regulatory effects on chromatin structure in LE-2 cells, forming a distinct long tail in the score distribution. **(B)** Plotting the DEEP+ scores (absolute values, y-axis) against GWAS risk Z-scores (x-axis, logarithmic scale) for each SNP revealed high-confidence loci with extreme risk associations and regulatory effects. These loci, representing the top 0.01% of all SNPs scanned, are highlighted as high-confidence candidates (data points in black). **(C)** The risk alleles (log Z-scores greater than 0) of the identified high-confidence candidate loci were more likely to decrease their local chromatin accessibility in LE-2, reflected by their reduced DEEP+ scores. **(D)** Linkage disequilibrium score regression (LDSC) analysis confirmed that the high-confidence loci were significantly enriched for prostate cancer heritability compared to loci with high GWAS risk Z-scores alone (without considering DEEP+ regulatory effects) or background genomic loci. Error bars represent standard deviation. **(E)** The high-confidence loci showed significant enrichment in gene promoter regions, revealing mutational convergence on gene promoters. P values were derived by Chi-square test. **(F)** Candidate genes with altered promoter accessibility due to the identified germline risk alleles (those with log Z-scores greater 0) are shown. Genes with decreased promoter accessibility (negative DEEP+ scores) are highlighted in blue, while genes with increased promoter accessibility (positive DEEP+ scores) are highlighted in red. Representative genes and functional categories (for those exhibiting reduced promoter accessibility) are also indicated. **(G)** Genes with perturbed promoters by germline risk alleles (open or close chromatin accessibility) display significantly increased gene dosage sensitivity. P values were derived from Wilcoxon rank-sum test. **(H)** The identified candidate genes showed co-occupancy of FOXA1 and AR at their promoters. ChIP-seq data for FOXA1 and AR binding were normalized based on benign prostate tissue samples. This co-occupancy underscores the convergence of germline risk onto FOXA1/AR-mediated regulon. All P values were corrected by the Benjamini-Hochberg method.

Establishing the model’s performance, we next leveraged DEEP+ to screen each germline allele for its regulatory impact in specific cell types. We trained DEEP+ models using cell type–specific open chromatin regions reconstructed from snATAC-seq data via pseudobulk analysis. Independent DEEP+ models were trained for each identified cell type, with particular emphasis on LE-2, which exhibited the strongest genetic predisposition to prostate cancer. Cross-validation confirmed high predictive accuracy for the LE-2 model on open chromatin regions from given genomic sequences (AUROC = 0.86 ± 0.02). We then applied DEEP+ to score each of the ~ 17 million SNPs in the prostate cancer GWAS dataset described above [10]. Each SNP was assigned a DEEP+ score quantifying its allelic effect on local chromatin accessibility, with positive and negative values indicating increased or decreased chromatin accessibility, respectively. To assess the magnitude of these regulatory perturbation, we examined the absolute DEEP+ scores (regardless of increasing or decreasing chromatin accessibility) for each SNP. We found that most germline variants were functionally neutral, exhibiting minimal impact on prostate chromatin structure (peaked at 0, **Fig 5A**). However, the long-tailed distribution revealed a distinct subset of germline variants that exerted strong regulatory influences in LE-2 (**Fig 5A**). Because the presence of regulatory effects alone does not necessarily indicate a relevance to prostate cancer susceptibility, we integrated the absolute DEEP+ scores with the prostate cancer GWAS risk Z-scores. This approach ensured identifying alleles that both significantly alter LE-2 chromatin accessibility and are directly linked to prostate cancer susceptibility. **Notably, GWAS variants were not pruned for linkage disequilibrium (LD) because each variant was independently assessed for its regulatory impact on gene expression.** Consequently, each GWAS variant was jointly represented by its risk Z-score from GWAS and its DEEP+ regulatory effect score, enabling mechanistic interpretation of how germline variants may influence gene regulation in LE-2 to modulate prostate cancer risk.

The joint distribution of these two scores for ~17 million GWAS variants is shown in **Fig 5B**. Our primary interest focused on the upper-right corner in **Fig 5B**, where variants not only exert strong regulatory effects in LE-2 (high absolute DEEP+ scores) but also display the highly significant prostate cancer GWAS risk (large |Z|-scores). These variants represented high-confidence functional germline variants with both mechanistic and statistical evidence for involvement in prostate cancer. To systematically prioritize these candidates, we employed a 2D Gaussian framework to model the genome-wide joint distribution of DEEP+ and GWAS risk Z-scores, which were statistically independent (the correlation coefficient R ≈ 0). We then selected loci with 2-D joint probability exceeding 0.90 (shown in black in **Fig 5B**). This practice prioritized 1,732 loci, representing approximately 0.01% of the ~ 17 million variants screened. These loci constitute a high-confidence set of germline variants exhibiting both prostate cancer risk association and putative functional consequences in the LE-2 regulatory landscape (**Fig 5B**).

Our variant prioritization above considered the magnitude of chromatin structure alterations (the absolute values of DEEP+ scores). For each variant, we then identified its risk-associated allele (log Z score>0), and observed that these risk alleles predominantly reduced chromatin accessibility (i.e., receiving negative DEEP+ scores, p = 3e-3, Wilcoxon rank-sum test, **Fig 5C**). Targeting these 1,732 top candidate loci (S3 Table), we next performed linkage disequilibrium score regression (LDSC) [41] and observed a striking 3,806-fold enrichment for prostate cancr risk relative to the genomic background (P = 2.52e-29). This enrichment far exceeded the more modest 73-fold risk enrichment observed when selecting loci solely by high GWAS risk scores (with the same risk Z-score threshold applied to the top candidate loci) without considering their regulatory allelic effects (Fig 5D). This contrast was expected due to that many high risk GWAS variants were merely driven by linkage disequilibrium proxies rather than direct biological activity, underscoring the importance of incorporating functional insights when interpreting GWAS signals. To gain deeper functional insights, we annotated genomic locations of the identified top candidate loci and found that 181 of the 1,732 loci (~10.4%) resided in gene promoters. This finding demonstrated a significant enrichment for promoter-associated variants, a trend absent when loci were selected solely based on GWAS risk Z-scores (Fig 5E). Interestingly, promoter enrichment further increased under more stringent thresholds (S4A Fig), highlighting the disproportionate representation of promoter-associated variants among the prioritized risk loci. Therefore, by integrating regulatory effects with GWAS risk, we were able to identify functional variants from the statistically associated loci. The pronounced promoter enrichment further indicates that disruption of LE-2 promoter activity is likely a recurrent mechanism underlying germline risk for prostate cancer.

We closely examined the 181 genes with affected promoters (**Fig 5E**). The magnitude of the alterations on their promoter accessibility is shown in **Fig 5F**, where at-risk alleles opening promoters (in red) or closing promoters (in blue) are indicated (S4 Table). One prominent example is *rs11781886* at the *NKX3–1* promoter locus, where the risk allele T was predicted to disrupt the chromatin accessibility in LE-2 cells (S4B Fig). Strikingly, genes with risk alleles that increase promoter accessibility were overwhelmingly enriched for oncogenic or prostate-cancer-upregulated genes. For example, *RPS3A* stands out as an extreme outlier, driven by a risk-associated variant that significantly enhanced its promoter accessibility. Overexpression of *RPS3A* induces tumor formation by suppressing apoptosis [42], and its expression is markedly elevated in prostate adenocarcinoma [43]. Similarly, *TTK*, a mitotic checkpoint kinase and recognized oncogene, is positively correlated with higher Gleason scores in prostate cancer [44,45]. Another mitosis-related gene, *TACC1*, which stabilizes microtubules during cell division and facilitates unchecked proliferation, is strongly up-regulated in hormone-refractory prostate cancer with distant metastasis [46]. In the context of hypoxia-driven pathways, *EGLN2* (egl-9 family hypoxia-inducible factor 2), a key regulator of cellular adaptation to low oxygen, shows elevated expression in prostate cancer, and its ubiquitination by the tumor suppressor *SPOP* constrains cancer cell growth [47]. Hypoxia-induced reprogramming of RNA splicing machinery up-regulates the oncogenic splicing factor *SRSF3*, which promotes prostate cancer progression [48,49]. Similarly, the mitochondrial protein *PGAM5*, which regulates oxidative stress responses and the ROS-induced cell death pathway (known as oxeiptosis), exhibits increased expression in high-grade prostate tumors and down-regulated following castration therapy [50–52]. Likewise, the metabolic gene *HMGCS1* is oncogenic in prostate cancer [53], and its overexpression significantly stimulates tumor cell growth [54]. The apoptosis regulator *DAXX* consistently displays up-regulation across cancers, including prostate cancer [55]. Additional cancer-associated genes affected by promoter-associated risk alleles (**Fig 5F**) include *CLIC1*, which facilitates prostate cancer proliferation and migration [56]; *MICA,* which enhances immune evasion mechanisms [57]; and *UHRF1 BP1,* which supports prostate tumor progression through epigenetic regulation [58]. Taken together, genes up-regulated in prostate cancer tend to harbor germline risk alleles that increase their promoter accessibility, thereby predisposing normal luminal epithelial cells to a cancer-prone state (**Fig 5F**, genes in red).

Likewise, genes with germline alleles reducing promoter accessibility (**Fig 5F**, genes in blue) tended to be down-regulated in prostate cancer or function as tumor suppressors. Representative genes in related functional categories are summarized in **Fig 5F**. We particularly note the well-known prostate tumor suppressor *NKX3–1* [59], which harbors a germline variant that markedly decreases its promoter accessibility, potentially attenuates its transcriptional activity. Several additional tumor suppressors were similarly affected by promoter-closing variants, including *BBC3* (*PUMA*), a pro-apoptotic gene that suppresses prostate cancer cell growth [60], and known tumor suppressors, *ATL3* [61] and *ING5* [62]. Promoter-closing variants further targeted genes essential for genome stability. *GTF2H1* and *ERCC6* regulate nucleotide excision repair [63,64], and their loss is expected to result in the compromised ability of cells in DNA repair, a hallmark of cancer progression. Additionally, *DNMBP*, which supports epithelial structure by regulating apical junctions [65,66], is consistently down-regulated at the protein level across human tumor types (the Human Protein Atlas) [67]. Reduced promoter accessibility in LE-2 may destabilize epithelial integrity and promote metastasis. We particularly note *E2F7*, whose promoter is occupied by p53 [68]. In response to DNA damage, p53-dependent up-regulation of E2F7 serves as a critical brake on proliferation by repressing cell cycle progression genes [68]. Therefore, germline variants that close the *E2F7* promoter likely obstruct p53 binding, compromising this critical checkpoint and resulting in uncontrolled proliferation. Taken together, the identified promoter-opening alleles tended to affect oncogenes, whereas promoter-closing alleles tended to target tumor suppressors. This directional disruption predisposes LE-2 cells to elevate cancer risk by compromising critical cellular processes such as DNA repair, apoptosis, and cell cycle regulation.

### The identified candidate genes are sensitive to dosage alterations

We hypothesized that if the identified germline variants contribute to tumorigenesis by disrupting gene regulation, then the affected genes would likely exhibit dosage sensitivity. To evaluate this, we utilized gene haploinsufficiency scores [69] as a proxy for dosage sensitivity. Haploinsufficient genes are known to be intolerant to dosage imbalance and can be toxic when overexpressed [70] due to the need for tightly controlled expression levels [71]. Our analysis revealed that the identified genes showed significantly higher dosage sensitivity compared to the genomic background (**Fig 5G**). This enrichment was especially pronounced among genes whose promoter accessibility was increased by germline risk alleles (P = 1.1e-3, Wilcoxon rank-sum test). These findings indicate that our identified allelic perturbations preferentially affect dosage-sensitive gene promoters, supporting their functional consequences to prostate cancer susceptibility.

### The at-risk germline alleles are convergent onto the AR-mediated regulon

Since the identified genes and their associated allelic effects were specific to the LE-2 subtype, which is characterized by the highest expression of the androgen receptor (AR, **Fig 2B** and **2D**), we investigated whether these genes, despite their involvement in diverse functional groups (**Fig 5F**), converge on AR-associated pathways, a hallmark of prostate cancer etiology [21,72]. In the normal prostate, AR recognizes its target genes through cooperation with the pioneering chromatin remodeler FOXA1, which opens chromatin to facilitate AR binding at prostate-specific loci. Disruption of FOXA1 guidance leads to promiscuous AR binding to androgen response elements, even in the absence of androgen, leading to aberrant transcriptional activation of oncogenic programs [73] and activating genes associated with castration-resistant prostate cancers (CRPC) [24]. Therefore, the delicate balance of transcriptional co-regulation by FOXA1 and AR defines the AR-mediated regulatory program in prostate cells, and perturbation of this equilibrium is a key driver of tumorigenesis [22].

Interestingly, the high-confidence candidate genes identified in this study, which are affected by germline variants in prostate cancer, displayed strong enrichment for FOXA1 targets based on ChIP-seq data from non-cancerous LHSAR cells [23], an immortalized prostate epithelial line expressing exogenous AR [74] (FDR = 8.23e-7, Methods and Materials). To validate these findings, we first confirmed that *FOXA1* is co-expressed with *AR* in LE-2 cells (S5 Fig). We then re-analyzed ChIP-seq data for FOXA1 and AR in benign human prostate samples [23] and observed significant co-binding peaks at the promoters of the identified genes (Fig 5H). This co-occupancy suggests that these promoters are likely targets of FOXA1-assisted AR recruitment, establishing prostate-specific AR-mediated regulatory networks. This co-binding was also evident for genes with promoters either opened or closed by germline variants (blue or red genes, Fig 5F) (S6 Fig). These observations imply that increased promoter accessibility likely hyperactivates AR function, while decreased accessibility likely impairs FOXA1/AR binding to prostate-specific genes. Together, these germline risk variants would dysregulate the FOXA1/AR equilibrium via remodeling promoter accessibility, potentially converting LE-2 cells into a cancer-prone state.

## Discussion

This study presents the first germline risk architecture of prostate cancer at single-cell resolution, unveiling the cellular basis of heritability that has remained elusive in literature. Whereas most existing studies have focused on somatic mutations, germline variants have largely been explored through GWAS, which, despite its utility, has struggled to translate statistical associations into functional mechanisms. By integrating GWAS with single-cell data, our study systematically partitioned germline risk across distinct cell types, enabling the identification of the cell populations most genetically susceptible to prostate cancer. Furthermore, our deep learning framework provided a novel approach to quantify the regulatory effects of individual alleles genome-wide, offering a scalable strategy to functionally interpret GWAS risk loci. Importantly, the germline risk loci and their target genes uncovered in our study converge on AR pathways, consistent with well-established somatic drivers of prostate cancer. This highlights a shared mechanism between germline and somatic alterations, emphasizing the pivotal role of AR signaling in prostate tumorigenesis. Overall, our findings provide critical cellular and molecular insights into the heritability of prostate cancer. This integration of genomics, single-cell analysis, and deep learning establishes a generalizable blueprint for dissecting germline predisposition across diverse cancer types.

Our single-cell analysis identified distinct luminal epithelial subtypes, and many well-known prostate cancer-associated genes are uniquely expressed in specific luminal subtypes (Fig2). This observation highlights that different luminal subtypes may collectively contribute to prostate cancer susceptibility and progression. Our findings were validated independently using GTEx dataset, which recapitulated the characteristics of luminal subtypes in the prostate. Notably, we examined published single-cell data from primary prostate tumors [28] but many of these luminal subtypes were not detectable. For instance, LE-2 cells showing high *LRRC7* expression were absent in primary prostate tumors (S7 Fig). This likely reflects the massive dysregulation in malignant luminal epithelial cells that disrupts normal cell-type identities. Therefore, sequencing normal prostate samples will not only provide a reference baseline for fine mapping mutations but also offer unique insights into the early events of tumor initiation. Although this study focused primarity on the LE-2 subtype, this does not preclude the contribution from other luminal subtypes or their progenitors (e.g., the club-like cells) through distinct mechanisms. Thus, the uncovered luminal subtypes warrant further investigation to elucidate their respective roles in tumor initiation, progression, and treatment responses.

Our study is particularly focused on the terminally differentiated luminal epithelial cells (LE-2), which exhibit the strongest enrichment of germline risk in prostate cancer. Thus, we propose that terminally differentiated LE-2 cells represent the cell of origin for germline predisposition in prostate cancer. In early studies, basal epithelial cells were considered the likely cell of origin in prostate cancer [75–77], and luminal progenitor cells have also been implicated [78,79]. Our findings suggest a developmental transition from luminal progenitors, including club cells, to terminally differentiated luminal epithelial subtypes prior to malignant transformation. This model is consistent with *in vivo* lineage-tracing studies that identified luminal epithelial cells as the likely cells of origin for prostate cancer [77], but further investigation is needed to delineate the full differentiation trajectory, particularly the transient club-cell state. Prostate cancer predominantly display a luminal phenotype and expresses luminal markers [79,80]. Our single-cell germline mapping now provides genetic evidence that the terminally differentiated luminal epithelial cells (the LE-2 subtype) is the cell type of origin in prostate cancer given the substantially amplified germline risk in this subtype (**Fig 3**). At the molecular level, the chromatin landscape of stem-like and progenitor cells is more poised or repressed [81,82], limiting the ability of germline variants to affect regulatory activity. Germline effects are expected to manifest when these regulatory elements are progressively activated in a more differentiated state [83–85]. This notion is well supported by our analysis, where germline risk progressively increased along the cell developmental trajectory, culminating in the terminally differentiated LE-2 cells (**Fig 3C**). Furthermore, mature luminal cells are exposed to various physiological stress, such as oxidative stress and inflammation, due to their metabolic activity and role in prostatic fluid secretion [86,87]. These stressors may amplify the regulatory effects of germline variants, thereby increasing susceptibility to malignant transformation. While our data showed that LE-2 cells as the primary mediators of germline risk in prostate cancer, we emphasize that this does not exclude the contributions from other cell types driven by somatic mutations, environmental stimulation or other pathogenic mechanisms.

Our study next leveraged our deep learning model to screen each germline allele across the genome for its potential regulatory effects in LE-2. Unlike most of the existing generalized models that learn DNA sequence patterns from heterogeneous datasets (such as local genomic features like ChIP-seq and chromatin accessibility) from different tissues, our framework is task-oriented and specifically tailored to prostate tissues and LE-2 cells by training on our own single-cell multiomic profiles. Thus, our design would help identify pathogenic alleles with cell-type-specific regulatory consequences instead of merely capturing conserved functional loci. By assessing allelic influences on gene regulation, we established mechanistic links between GWAS associations and underlying functional consequences (**Fig 5B**). This approach thus offers a powerful framework to derive functional insights from statistical associations and underscores the substantial role that the noncoding genome may play in prostate cancer etiology. Notably, these findings appear to contrast with previous interpretations. While it is commonly accepted that noncoding regulatory variants drive complex diseases [88], the recent Pan-Cancer Genome Analysis proposed that noncoding regulatory mutations may not significantly influence cancer development [89]. However, the conclusion relied solely on comparing raw mutation counts between tumors and controls, without considering the functional impact of individual variants on gene regulation. By incorporating a deep-learning-based framework to move beyond statistical associations and directly quantify the regulatory consequences of every single allele, our study paves the way for a more refined and mechanistic understanding of the genetic architecture of cancer.

For the identified germline alleles in prostate cancer, we identified a prevalent mechanism of perturbing gene promoters. Examination of individual affected genes revealed a strong pattern: alleles that increase promoter accessibility tend to affect oncogenes, whereas alleles that decrease promoter accessibility predominantly target tumor suppressors. Additional regulatory elements such as enhancers and silencers influenced by genetic perturbations likely play significant roles in prostate cancer susceptibility. Definitive functional interpretation of these elements as oncogenic or tumor-suppressive will require future experimental validation, such as CRISPR-based genome editing in prostate-relevant cell types. Thus, our observations suggest that germline risk variants adopt a tumor-like epigenomic configuration on LE-2 cells, preconditioning them toward a pre-malignant state. Notably, many of the re-programmed promoters belong to genes involved in DNA repair (**Fig 5F**), implying that germline perturbations could diminish cells genome stability and sensitize cells to the accumulation of additional somatic alterations, thereby accelerating malignant progression. We particularly highlight that the genes affected by risk germline variants are convergent onto the FOXA1/AR-mediated regulon, but the direct allelic effects on FOXA1 binding affinity will require experimental validation through CRISPR base editing or massive parallel reporter assays. Our finding is in consistency with prior work showing that non-coding mutations accumulating in prostate cancer are likely perturb the binding affinity of FOXA1 as well as alter the AR cistromes [90]. Moreover, extensive studies [22–24,73,91–98], including our previous work [37], demonstrated that somatic mutations in prostate cancer frequently converge on the FOXA1/AR pathways. Thus, regardless of mutation origin, perturbation of the FOXA1/AR-mediated regulatory program emerges as a unifying mechanism that drives prostate tumor formation and progression.

Germline variants have traditionally been considered as having weak effect sizes. However, the effect sizes are derived from allele frequency differences between case and control populations, and may therefore underestimate the biological impact of individual alleles on gene regulation (e.g., the outlier affecting *RPS3A* in **Fig 5F**). Even if single variants have weak regulatory effects, each individual typically carries ~4 million germline variants, and the cumulative impacts would substantially predispose individuals to cancer. Thus, identifying germline mutations and genetic predisposition to prostate cancer has significant implications for early detection and intervention. Moreover, because the germline variants we identified converge on the AR-mediated regulon, our findings raise the possibility that epigenetic therapies, including chromatin-modifying agents or AR-targeting drugs, could be tailored to reverse or mitigate the tumor-like epigenomic configuration in genetically high-risk individuals prior to malignant transformation.

In summary, our study, for the first time, revealed a key cell type mediating germline risk in prostate cancer and uncovered high-confidence loci in promoters of genes involved in AR-mediated regulons. This study offers a unifying framework for understanding prostate cancer heritability and establishes a direct connection between inherited genetic risk and epigenetic priming of tumorigenesis, paving the way for innovative screening, diagnosis, and therapeutic intervention aimed at intercepting cancer at its earliest stages.

## Methods and materials

### Ethics statement

Normal human prostate gland tissues were collected from two deceased donors through the ENCODE Consortium under the existing protocols met the relevant IRB standards. All materials from decedents were fully de-identified. The study was deemed not to involve human subjects and did not require additional IRB approval.

### Nuclei Isolation from normal prostate tissues

Normal prostate gland tissues were collected from two deceased donors with written informed consent. Samples were snap-frozen according to GTEx protocols [26] prior to single-nuclei isolation. Nuclei extraction was performed with slight modifications to previously described methods [99]. Briefly, flash-frozen tissues were mechanically ground on dry ice, and 30–50 mg of the ground tissue was transferred to a pre-chilled 7 mL PYREX Dounce homogenizer (Corning Life Science) containing 2 mL of cold homogenization buffer (250 mM sucrose, 0.3% NP40, 5 mM MgCl2, 25 mM KCl, 10 mM Tris pH 7.8, 1 × protease inhibitors (Roche, cOmplete), and 0.6 U/µL Ribolock RNase inhibitor (Thermo Fisher)). After homogenization, debris and connective tissues were removed using a 40 μm cell strainer (pluriSelect). Intact nuclei were purified using a three-layered OptiPrep Iodixanol (Sigma-Aldrich) gradient (25%, 30%, and 40% from top to bottom). Following centrifugation at 3000 r.c.f. for 30 minutes in a swinging-bucket centrifuge (Eppendorf 5810R), nuclei bands visible at the 30%–40% interface were collected. Nuclei purification and integrity were verified under a microscope, and approximately 10,000 nuclei from each sample were used as input for Chromium Next GEM Single Cell Multiome ATAC + Gene Expression (10x Genomics Inc.).

### Single-nucleus multiome library construction and sequencing

Isolated nuclei were processed for single-nucleus multiome library construction using the Chromium Next GEM Single Cell Multiome ATAC & Gene Expression kit (10x Genomics Inc.), following the manufacturer’s protocols. Libraries for snATAC-seq and snRNA-seq were sequenced on the Illumina NovaSeq 6000 platform with parameters recommended by the manufacturer. Raw sequencing signals (BCL format) were demultiplexed into FASTQ files using the CellRanger-ARC suite (v2.0.0, 10x Genomics Inc.). Each snATAC-seq or snRNA-seq library yielded an average of 250 million paired-end reads. CellRanger-ARC was also used for barcode calling, read alignment, and quality assessment using the human reference genome (GRCh38 v3.0.0), following the protocols described by 10X Genomics (https://support.10xgenomics.com/single-cell-gene-expression/software/pipelines/latest/advanced/references).

### Quality control and doublet detection

Data quality for each sample is summarized in S1 Table. The CellRanger-ARC suite (v2.0.0) was used for initial barcode calling and quality assessment, retaining only high-quality sequenced nuclei. Nuclei passing the following criteria were included in downstream analyses: (a) Gene expression counts (gex_n_counts) between 1,000 and 50,000. (b) Detection of >400 genes (n_genes). (c) Less than 20% mitochondrial gene expression (percent_mito). (d) Total ATAC fragment counts (atac_n_counts) between 1,000 and 100,000. (e) Transcription Start Site (TSS) enrichment score >1. (f) Nucleosome signal strength <2. Among the retained nuclei, we identified potential doublets by Scrublet, and we excluded nuclei that were designated as potential doublets by Scrublet from our downstream analyses [100].

### Data Integration and batch alignment

For snATAC-seq, open chromatin peaks were identified in individual samples using MACS2 v2.2.7 [101]. Peaks from all samples were unified into genomic intervals, excluding regions blacklisted by ENCODE. The ATAC-seq fragments in peaks were counted as an input matrix for downstream analysis. Peak-by-nuclei count matrices were integrated using reciprocal LSI projection functions in the Signac package [102]. For snRNA-seq, gene-by-nuclei count matrices were integrated using reciprocal PCA projections with Seurat. Data normalization, scaling, and variable feature detection were performed following best practices [102,103].

### Data integration and batch alignment

For snATAC-seq analysis, open chromatin region peaks were called on individual sample using MACS2 v2.2.7 [101]. Peaks from all samples were unified into genomic intervals, and the intervals falling in the ENCODE blacklisted regions were excluded from downstream analyses. The ATAC-seq fragments in peaks were counted as the input matrix for downstream analysis. The peak-by-nuclei count matrices from each sample were integrated by reciprocal LSI projection functions using Signac package. For snRNA-seq data, the gene-by-nuclei count matrices for all nuclei passing quality control were integrated by reciprocal PCA projections between different samples using Seurat. Normalization, data scaling and variable features detection were subsequently performed by Signac/Seurat following the best practice described in Stuart et al.[102,103].

### Pseudotime trajectory inference

Pseudotime analysis was conducted with Monocle 3 [104]. Pseudotime values for individual cells were projected onto a two-dimensional UMAP embedding and smoothed using neighboring cell information. Differentiation trajectories were visualized with directional arrows that represent the maximum differentiation potential to illustrate progression from initial to adjacent cell states.

### Identification of cell type-specific genetic risk

SCAVENGE [36] was implemented to integrate snATAC-seq data with GWAS data [10] to quantify cell type-specific genetic risk for prostate cancer. GWAS summary statistics were obtained from Schumacher et al. [10] via the GWAS Catalog (accession: GCST006085). All GWAS variants were subjected to genome-wide conditional and joint association analyses using GCTA-COJO [105]. Sentinel loci were identified as those with the highest posterior probability (PP) within each linkage disequilibrium (LD) block. For each cell, the PP values of all sentinel SNPs overlapping open chromatin regions were aggregated to compute a trait-relevant score (TRS) for prostate cancer. Permutation tests were performed to assess the robustness of genetic vulnerability at the single-cell level, providing confidence in the TRS calculations. Cells with TRS values in the top 5% were classified as risk associated. To further refine the analysis, additional cell type-specific permutation tests using label shuffling were conducted to identify significant enrichment of risk-associated cells within specific cell types. The normalized proportion of risk-associated cells in each cell type was then calculated and visualized, offering insights into cell type-specific genetic predisposition to prostate cancer. All calculations were implemented by SCAVENGE [34].

### The DEEP+ model

The DEEP+ model was extended from our earlier DEEP model [106]. In this study, we trained the model using bulk prostate ATAC-seq data as we performed in our previous work [106] or using the ATAC-seq data in this study only from the LE-2 cells. Pseudobulk ATAC-seq peaks for each cell type were called using MACS2 v2.2.7 [101]. A residual-network-based DEEP+ neural network [106,107] was trained to capture sequence contexts defining cell type-specific open chromatin. Cross-validation (20-fold) was performed to optimize the model, and a held-out chromosome was used for independent validation. The model was applied to quantify the allelic effects of GWAS variants on chromatin accessibility in different cell types. Model performance was assessed using ROC curves on the held-out test chromosome.

### eQTL analysis

Prostate gland eQTLs were identified using CAVIAR fine-mapping data from GTEx Analysis V8 (dbGaP Accession phs000424.v8.p2) [38]. Variants were categorized based on their posterior probability (PP) as follows: those with PP > 0.9 were designated as high-confidence eQTLs (HC-eQTLs), those with PP < 0.1 were classified as non-eQTLs, and variants with PP values falling between these thresholds were considered low-confidence eQTLs (LC-eQTLs).

### Identification of DEEP+ candidate loci and genes

Variants were prioritized using a two-dimensional Gaussian model that integrated genome-wide DEEP+ scores and GWAS Z scores. To ensure consistency and account for score magnitude, the absolute values of the scores were logarithmically transformed prior to analysis. Variants with a joint probability exceeding 0.90 were designated as candidate loci. Associated genes were subsequently annotated using Homer [108], enabling the identification of regulatory elements and promoter regions linked to these high-confidence variants.

### Partitioned heritability analysis

Partitioned heritability analysis was implemented by LD score regression as previously described [109,110]. Briefly, heritability was partitioned across loci grouped into three categories: (A) prioritized candidate loci (n = 1,732) receiving high DEEP+ and GWAS Z-scores (minimum Z = 2.47); (B) genetic loci with GWAS Z-scores exceeding the threshold from group A (Z = 2.47) but showing insignificant regulatory effects; and (C) all remaining loci in GWAS datasets. Heritability enrichment was estimated by using the LDSC software package [41,110]

### Gene ontology and dosage sensitivity analysis

Enriched functional categories for genes linked to risk alleles were identified using Enrichr [111]. FDR values were computed using the Benjamini-Hochberg correction. Dosage sensitivity was approximated using the haploinsufficiency scores [69], and the Wilcoxon rank-sum test was used to determine statistical significance.

### ChIP-seq analysis

ChIP-seq data AR and FOXA1 in benign prostate tissue samples were downloaded from GEO (accession # GSE56288). For a given list of genes, occupancy of FOXA1 or AR at the candidate promoters was quantified by averaging the ChIP-seq signals intensity from multiple bigwig files before aligning the 6Kb flanking regions of the transcriptional starting sites (TSS). Enrichment of the ChIP-seq signals were compared to the genome background and visualized using deepTools and [112].

## Supporting information

S1 Fig Optimization of clustering parameters for Louvain-based cell type identification. Line plots depict metrics used to evaluate clustering performance across a range of Louvain resolution values: (**A**) mean silhouette scores across clusters, (**B**) mean within-cluster inertia, and (**C**) total number of clusters identified. The clustering resolution selected for downstream analyses is highlighted in red.
(PDF)

S2 Fig **(A-D)** Expression of club cell markers *CP, WFDC2, PIGR, and ELF3* on the UMAP. (**E-F**) Reconstructed pseudotime trajectory of luminal epithelium differentiation from BE to terminal LE states LE-4 (E) and LE-5 (F). Pseudotime of individual cells of each lineage is color-coded.
(PDF)

S3 Fig Schematic of DEEP+ model for allelic specific chromatin activity analysis. Input DNA sequences are first one-hot encoded, where each nucleotide (A, T, C, G) is represented as a binary vector. The encoded sequences are passed through multiple residual blocks, each consisting of a series of convolutional layers followed by batch normalization and activation functions. Skip connections (labeled as “Identity”) add the input of each residual block to its output to facilitate gradient flow and improve training stability. The final representation is passed through a fully connected layer to generate the output. This architecture enables effective learning of complex regulatory syntax from DNA sequences.
(PDF)

S4 Fig **(A)** Bar plots showing the percentage of promoter SNPs with different thresholds on joint probability (p = 0.05-0.95) from the 2D Gaussian distribution. Risk alleles with extreme regulatory functions are enriched in promoter regions. **(B)** Genome track showing one candidate promoter SNP *rs11781886* at the *NKX3–1* promoter locus with top DEEP+ score. Prostate cancer risk allele T was predicted to disrupt chromatin accessibility.
(PDF)

S5 Fig **(A)** The concordant dynamics of *FOXA1* and *AR* expression along the transition from BE (basal epithelium) to LE-2 (luminal epithelium 2). (**B-C**) Expression of *AR* (B) and *FOXA1*(C) on UMAP embeddings of snRNA-seq indicates the co-expression of two genes across all luminal epithelial cells.
(PDF)

S6 Fig (**A-B**) Co-occupancy of AR (**A**) and FOXA1 (**B**) at the candidate promoters in benign prostate tissues across six donors identified by ChIP-seq. Candidate promoters that are closed or opened by the identified risk alleles were highlighted in dark or light blue.
(PDF)

S7 Fig (**A-C**) UMAP projection showing *LRRC7* expression in single-cell/nucleus data from (**A**) normal prostate gland in this study, (**B**) normal prostate gland in GTEx data, and **C**) primary prostate tumors in Song et al.
(PDF)

S1 Table
Quality measurement of snMultiome data in normal prostate gland tissues.
(XLSX)

S2 Table List of identified marker genes for cell type/subtypes.
(XLSX)

S3 Table List of identified candidate risk loci showing strongest regulatory effects on local chromatin structure.
(XLSX)

S4 Table
Summary of functional categories of genes associated with candidate risk loci located in promoter regions.
(XLSX)
