# Supplementary figures and images for: Decoding the germline genetic architecture of prostate cancer at a single cell resolution
Source: PLoS Genet. 2025 Dec 30;21(12):e1011975. doi: 10.1371/journal.pgen.1011975 (PMC12753072; doi:10.1371/journal.pgen.1011975)

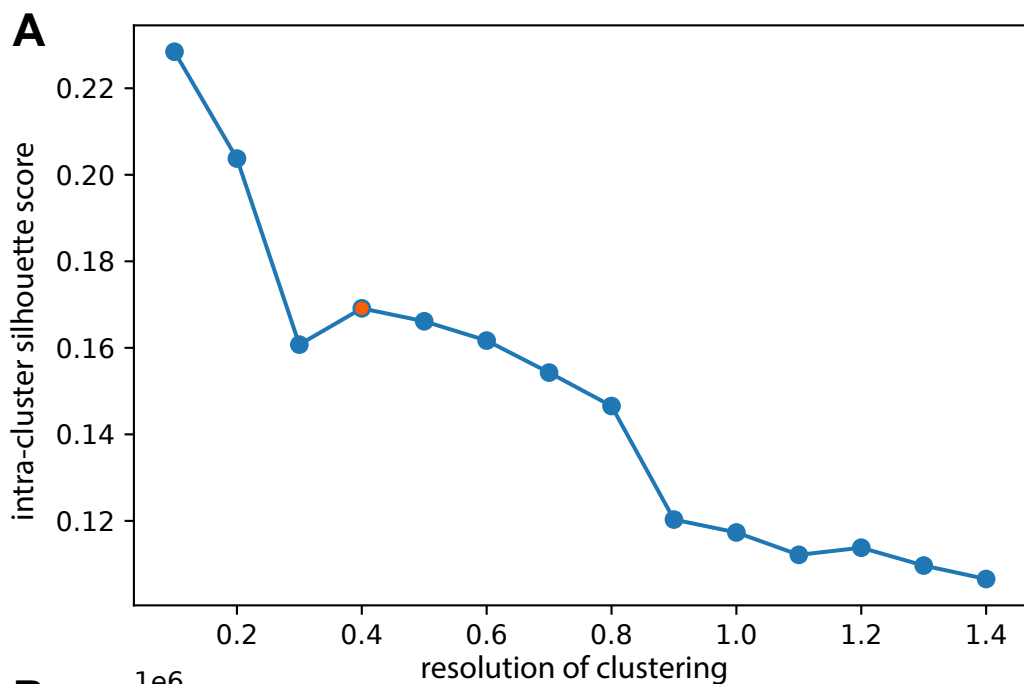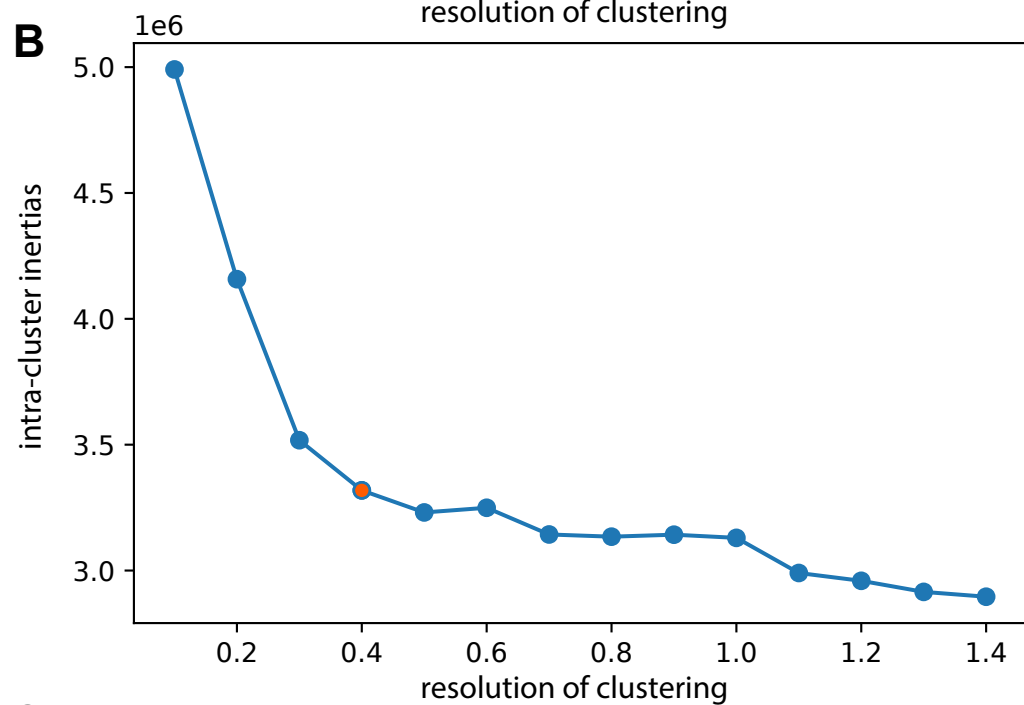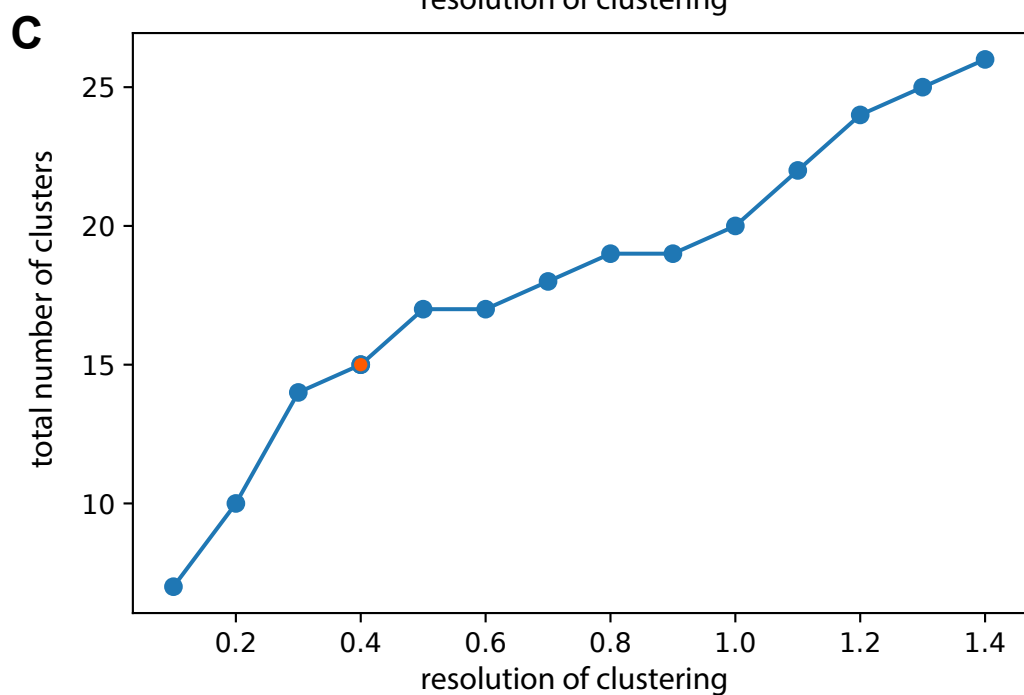

**Figure S1**

Supplement: S1 Fig — Line plots depict metrics used to evaluate clustering performance across a range of Louvain resolution values: (A) mean silhouette scores across clusters, (B) mean within-cluster inertia, and (C) total number of clusters identified. The clustering resolution selected for downstream analyses is highlighted in red. (PDF) [file pgen.1011975.s001.pdf]

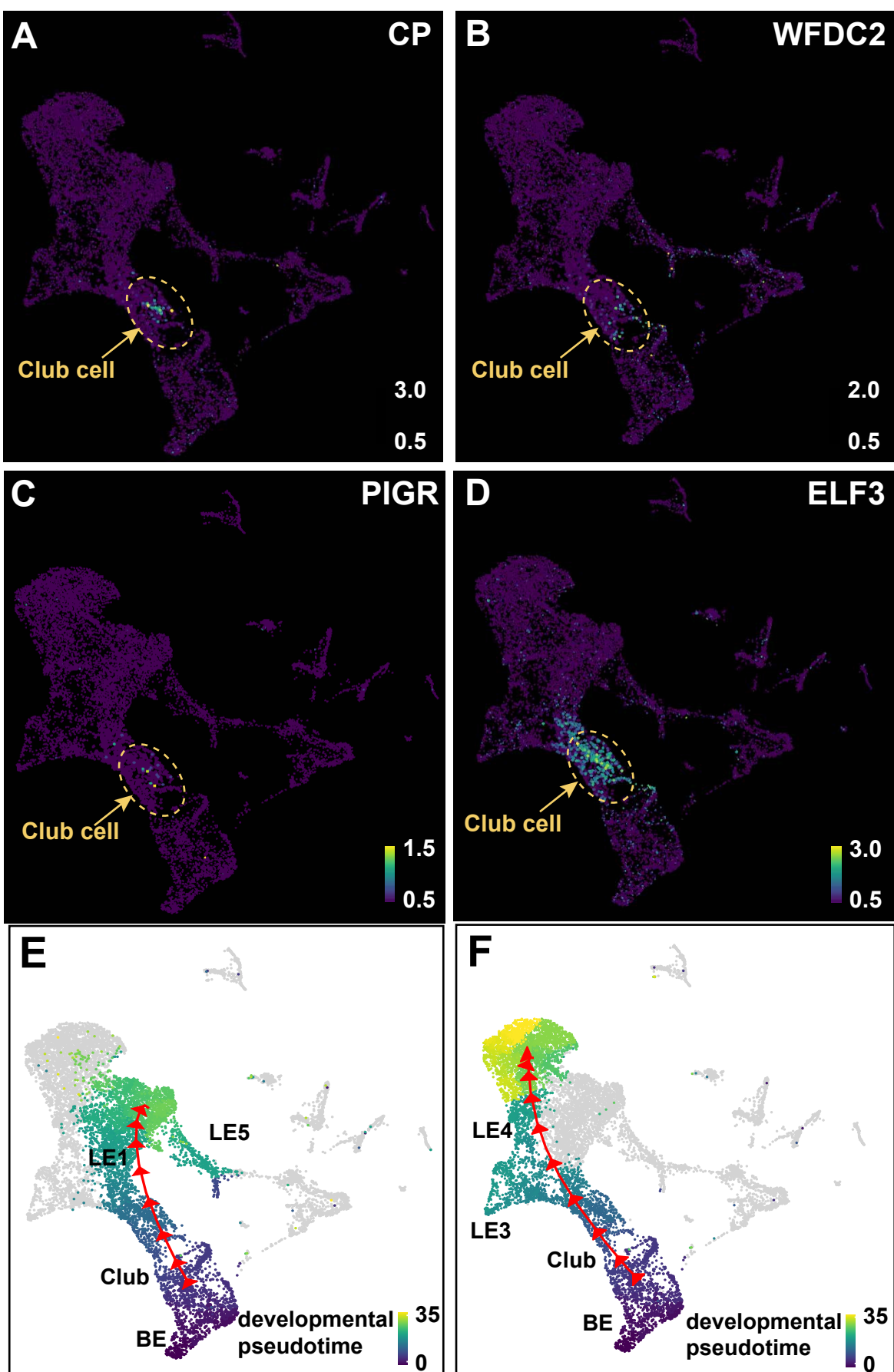

**Figure S2**

Supplement: S2 Fig — (A-D) Expression of club cell markers CP, WFDC2, PIGR, and ELF3 on the UMAP. (E-F) Reconstructed pseudotime trajectory of luminal epithelium differentiation from BE to terminal LE states LE-4 (E) and LE-5 (F). Pseudotime of individual cells of each lineage is color-coded. (PDF) [file pgen.1011975.s002.pdf]

... A A T C C T G ...

|   |   |   |   |   |   |   |   |   |
|---|---|---|---|---|---|---|---|---|
| A | 1 | 1 | 1 | 0 | 0 | 0 | 0 | 0 |
| T | 0 | 0 | 0 | 1 | 0 | 0 | 1 | 0 |
| C | 0 | 0 | 0 | 0 | 1 | 1 | 0 | 0 |
| G | 0 | 0 | 0 | 0 | 0 | 0 | 0 | 1 |

one-hot encoding

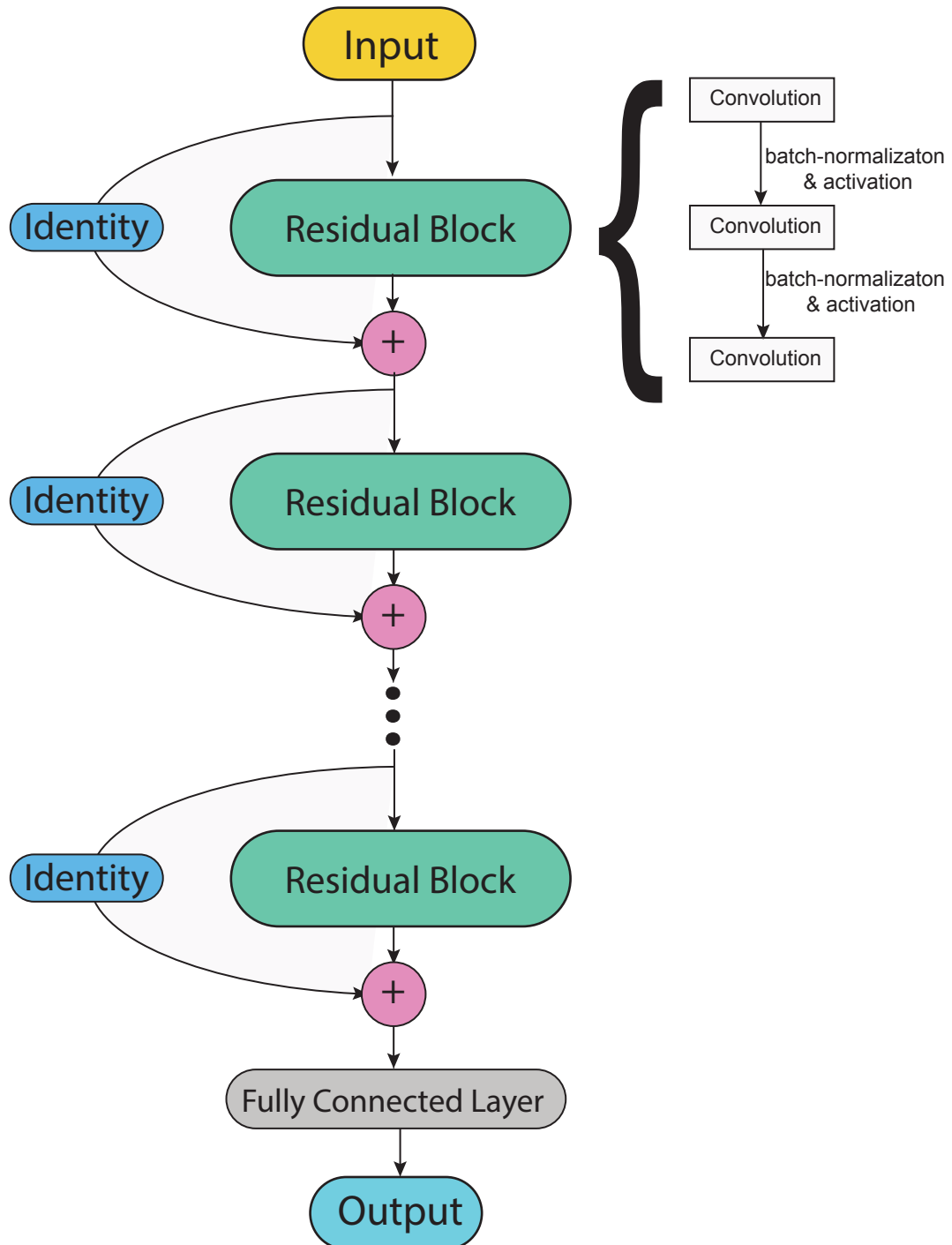

Figure S3

Supplement: S3 Fig — Input DNA sequences are first one-hot encoded, where each nucleotide (A, T, C, G) is represented as a binary vector. The encoded sequences are passed through multiple residual blocks, each consisting of a series of convolutional layers followed by batch normalization and activation functions. Skip connections (labeled as “Identity”) add the input of each residual block to its output to facilitate gradient flow and improve training stability. The final representation is passed through a fully connected layer to generate the output. This architecture enables effective learning of complex regulatory syntax from DNA sequences. (PDF) [file pgen.1011975.s003.pdf]

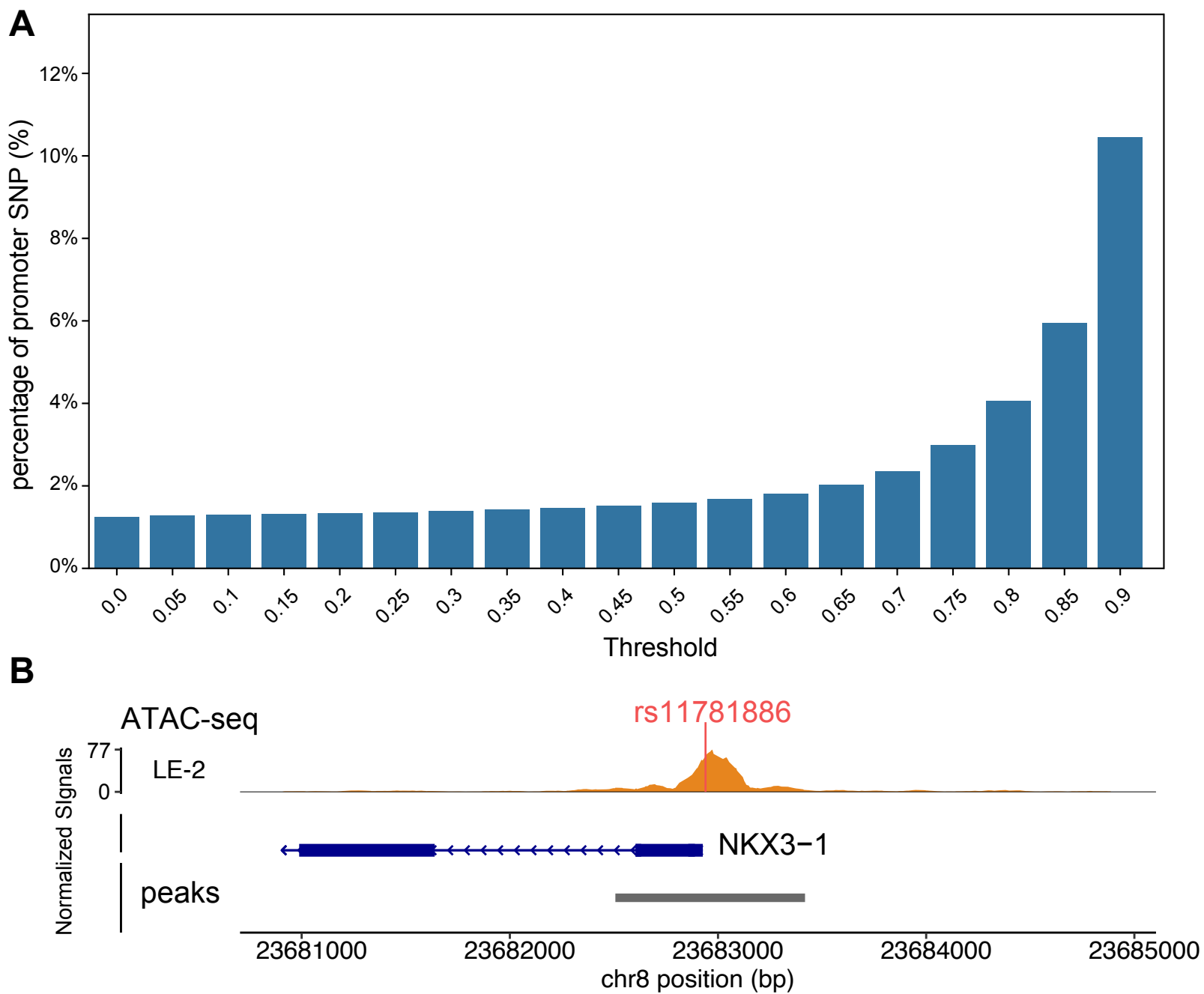

**Figure S4**

Supplement: S4 Fig — (A) Bar plots showing the percentage of promoter SNPs with different thresholds on joint probability (p = 0.05-0.95) from the 2D Gaussian distribution. Risk alleles with extreme regulatory functions are enriched in promoter regions. (B) Genome track showing one candidate promoter SNP rs11781886 at the NKX3–1 promoter locus with top DEEP+ score. Prostate cancer risk allele T was predicted to disrupt chromatin accessibility. (PDF) [file pgen.1011975.s004.pdf]

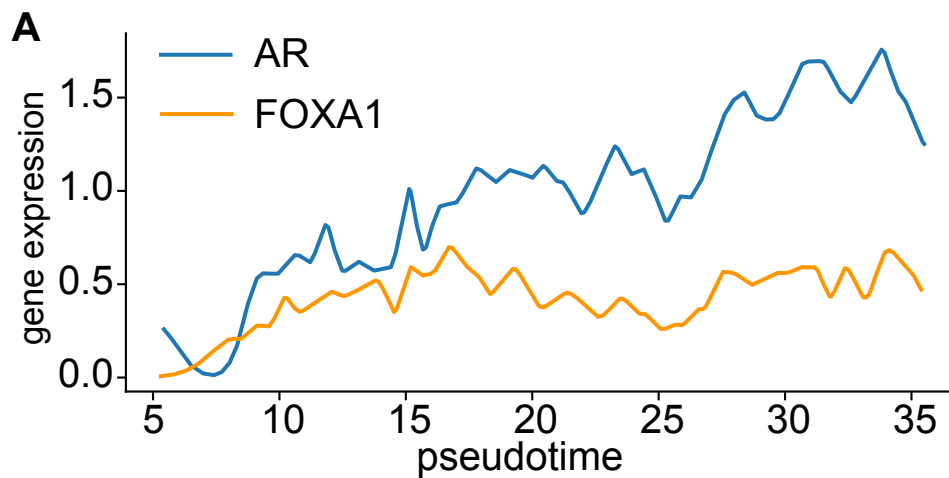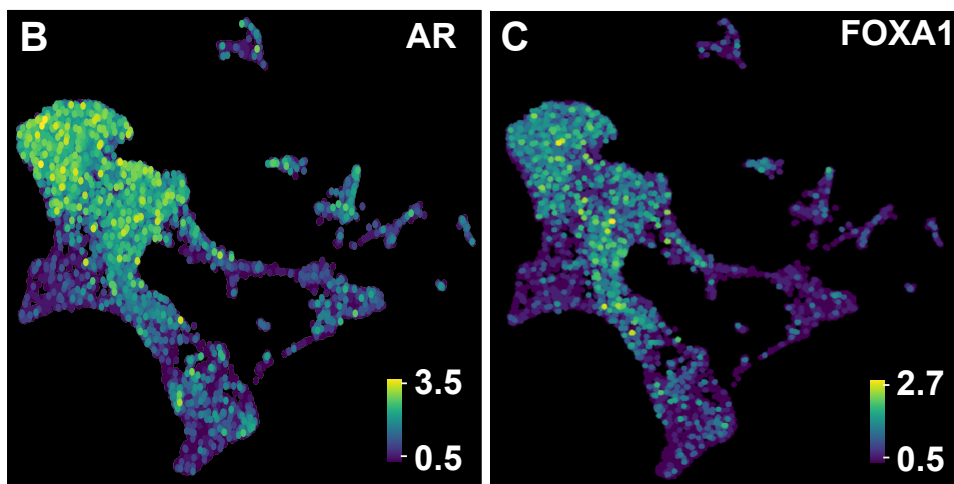

**Figure S5**

Supplement: S5 Fig — (A) The concordant dynamics of FOXA1 and AR expression along the transition from BE (basal epithelium) to LE-2 (luminal epithelium 2). (B-C) Expression of AR (B) and FOXA1(C) on UMAP embeddings of snRNA-seq indicates the co-expression of two genes across all luminal epithelial cells. (PDF) [file pgen.1011975.s005.pdf]

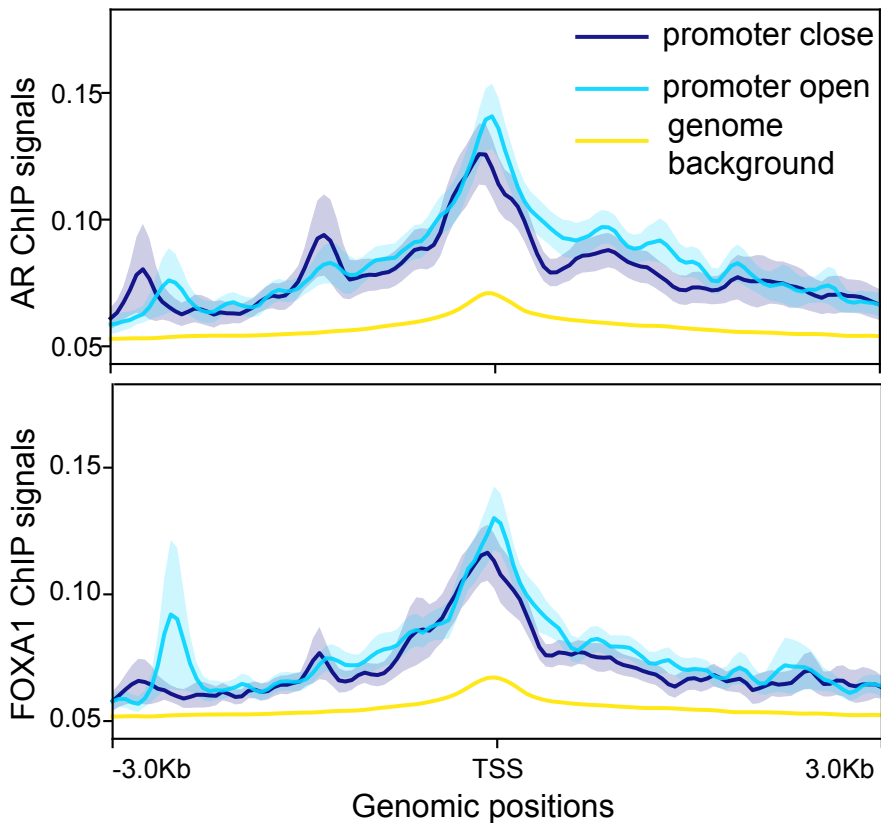

**Figure S6**

Supplement: S6 Fig — (A-B) Co-occupancy of AR (A) and FOXA1 (B) at the candidate promoters in benign prostate tissues across six donors identified by ChIP-seq. Candidate promoters that are closed or opened by the identified risk alleles were highlighted in dark or light blue. (PDF) [file pgen.1011975.s006.pdf]

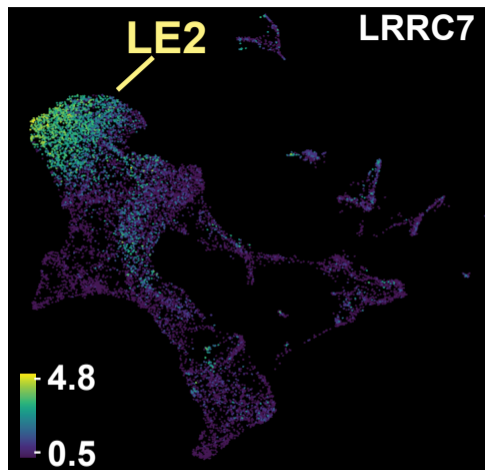

this study

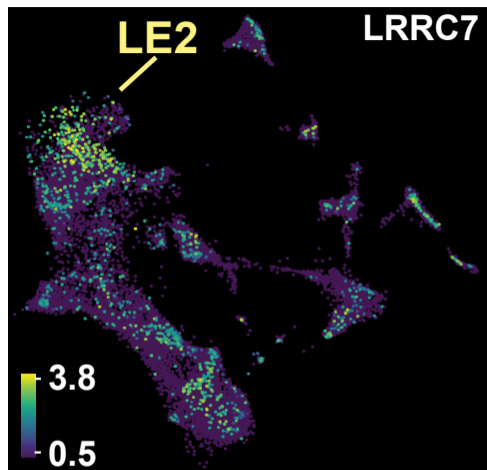

Eraslan et al. (GTEx)

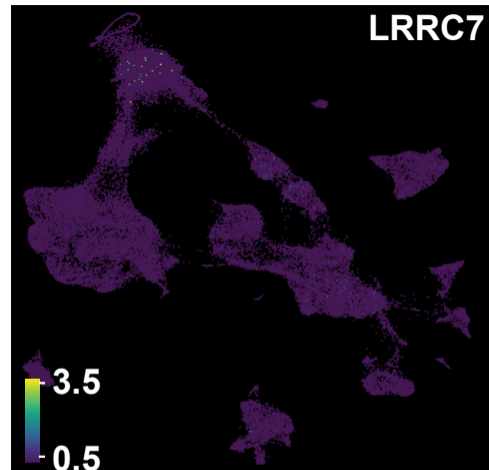

Song et al.

Figure S7

Supplement: S7 Fig — (A-C) UMAP projection showing LRRC7 expression in single-cell/nucleus data from (A) normal prostate gland in this study, (B) normal prostate gland in GTEx data, and C) primary prostate tumors in Song et al. (PDF) [file pgen.1011975.s007.pdf]
